# Out of Balance—Systemic Iron Homeostasis in Iron-Related Disorders

**DOI:** 10.3390/nu5083034

**Published:** 2013-08-02

**Authors:** Andrea U. Steinbicker, Martina U. Muckenthaler

**Affiliations:** 1 Department of Anesthesiology, Intensive Care and Pain Medicine, University Hospital Muenster, University of Muenster, Muenster 48149, Germany; 2 Department of Pediatric Oncology, Hematology and Immunology, University of Heidelberg, Heidelberg 69120, Germany; E-Mail: martina.muckenthaler@med.uni-heidelberg.de; 3 Molecular Medicine Partnership Unit (MMPU), Heidelberg 69120, Germany

**Keywords:** iron regulation, hepcidin, anemia, iron overload

## Abstract

Iron is an essential element in our daily diet. Most iron is required for the *de novo* synthesis of red blood cells, where it plays a critical role in oxygen binding to hemoglobin. Thus, iron deficiency causes anemia, a major public health burden worldwide. On the other extreme, iron accumulation in critical organs such as liver, heart, and pancreas causes organ dysfunction due to the generation of oxidative stress. Therefore, systemic iron levels must be tightly balanced. Here we focus on the regulatory role of the hepcidin/ferroportin circuitry as the major regulator of systemic iron homeostasis. We discuss how regulatory cues (e.g., iron, inflammation, or hypoxia) affect the hepcidin response and how impairment of the hepcidin/ferroportin regulatory system causes disorders of iron metabolism.

## 1. Introduction

**Figure 1:**
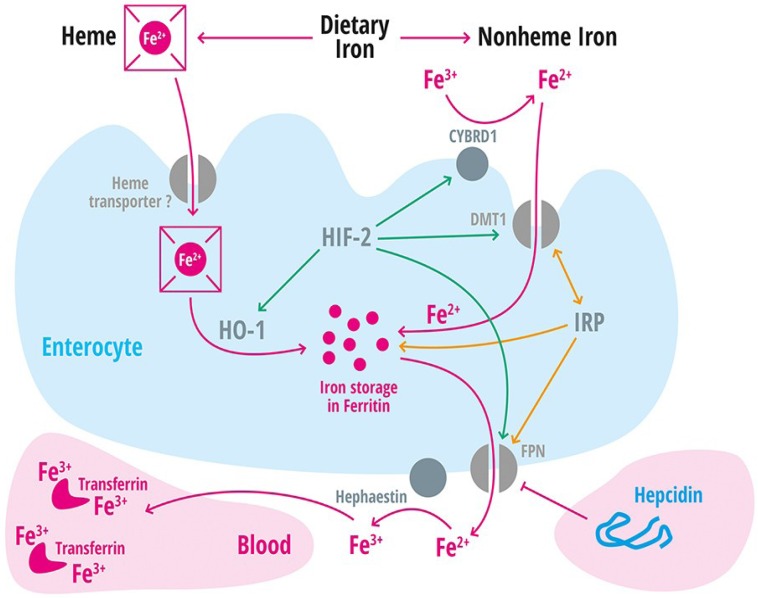
Iron absorption in the intestine. In the human diet, iron is present as heme or nonheme iron. Absorption of heme iron (Fe^2+^) is incompletely understood and likely mediated by a heme transporter. Intracellularly, iron is released from heme by hemoxygenase-1 (HO-1). Nonheme iron (Fe^3+^) is reduced by the membrane-associated ferric reductase CYBRD1 (DCYTB) for transport into the intestinal enterocyte by the divalent metal transporter (DMT1). Within the enterocyte iron can be stored in ferritin or exported into the blood stream by the iron exporter ferroportin (FPN1, SLC40A1). FPN expression is controlled by hepcidin. Hephaestin, a multicopper oxidase is required to incorporate two Fe^3+^ into one transferrin molecule (Tf). Hypoxia inducible factor 2 (HIF-2) controls CYBRD1, DMT1, FPN, and HO-1 mRNA expression (depicted in green) and iron regulatory proteins (IRPs) post-transcriptionally control the expression of DMT1, ferritin, and FPN (depicted in orange).

Iron is an essential nutrient and a potential toxin, and therefore its availability must be tightly controlled. It is a critical component of heme groups, iron-sulfur cluster-containing proteins, and of enzymes involved in mitochondrial respiration and DNA synthesis and thus, plays an important role in cellular metabolism, survival and proliferation. Iron deficiency causes anemia, a major public health concern affecting up to a billion people worldwide [1]. However, iron is also potentially toxic. It reacts with oxygen to generate reactive oxygen species (ROS), which trigger cell damage. Iron metabolism in mammals is balanced by three regulatory systems: one predominantly controls cellular iron metabolism through iron regulatory proteins (IRPs) that bind iron responsive elements (IREs) in regulated mRNAs [2] (Figure 1). The other regulatory system functions systemically and relies on the hormone hepcidin and the cellular iron exporter ferroportin (FPN1). In addition, the hypoxia inducible factors (HIFs) control transcription of numerous genes that maintain iron metabolism [3]. This review will focus on the regulation of systemic iron homeostasis by the hepcidin/ferroportin regulatory circuitry and its impairment in disease.

A healthy adult contains about 3–5 g of iron, which corresponds to approximately 45–60 mg/kg body weight. Two third of the total body iron content is bound to erythrocyte hemoglobin, whereby approximately 0.5 mg iron is contained in 1 mL blood (in a setting with a hemoglobin concentration of 15 g/100 mL). Twenty milligrams (20 mg) of iron are required daily for *de novo* hemoglobin synthesis, which is predominantly made available by iron recycling from aging erythrocytes. Another 5 mg of iron is exchanged daily within iron containing enzymes and iron stores [4]. Because there is no regulated pathway for iron excretion, only the small amount of iron (1–2 mg) that is lost due to bleeding, sweating, skin desquamation, or urinary excretion, is compensated for by iron absorbed from the diet. Elevated iron requirements during pregnancy or after bleeding are maintained by increasing iron absorption.

In the vegetarian diet, iron is predominantly present in its oxidized (Fe^3+^) state. For transport into the intestinal enterocyte by the divalent metal transporter (DMT1), it must be reduced by the membrane-associated ferrireductase CYBRD1 (DCYTB) (Figure 1). Additional enzymes may play a role in reducing iron, as Cybrd1 knock-out mice do not show an iron phenotype under steady-state conditions [5]. However, under hypoxic conditions, Cybrd1 knock-out mice show abnormal erythopoiesis and decreased splenic iron stores suggesting that Cybrd1 is required to allow for elevated iron requirements under stress conditions [6].

In the meat diet, iron is predominantly found in the heme form. A heme transporter may be involved in heme transport into the enterocyte, but its identity is currently not resolved (Figure 1). Intracellularly, iron is released from heme by hemoxygenase-1 (HO-1).

Within the enterocyte, iron is stored in ferritin and an enterocyte-specific role for ferritin in controlling iron absorption has been identified in mice [7]. How iron reaches the basolateral membrane is currently not completely understood. Iron export into the blood stream requires the iron exporter FPN1 (SLC40A1), which is regulated by hepcidin, HIF-2a [8], and by IRPs [9,10]. Hephaestin, a multicopper oxidase homologous to ceruloplasmin, is necessary to incorporate Fe^3+^ in the plasma protein transferrin (Tf) (Figure 1) [11]. Diferric transferrin circulates in the blood and provides iron to most cells of the body. In addition, transferrin-bound iron (Tf-Fe_2_) is a major indicator and determinant of systemic iron homeostasis. Iron saturation of transferrin is predominantly determined by the amount of iron: (1) absorbed from the intestine; (2) recycled from senescent red blood cells and released by macrophages; and (3) utilized for erythropoiesis [12].

Systemic iron fluxes are controlled by the hepatic peptide hormone hepcidin [12]. Hepcidin is mainly synthesized in hepatocytes and circulates in the plasma bound to alpha 2-macroglobulin [13]. Other cell types and organs, such as monocytes [14], macrophages [15], heart [16], kidney [17], brain [18], and adipose tissue [19], also produce hepcidin, albeit to much lesser extent. Hepcidin controls surface expression of the iron exporter FPN1 in enterocytes [20], macrophages and hepatocytes which express high levels of FPN1. It binds to FPN1, triggers its internalization, ubiquitination and degradation [21,22]. At the same time, hepcidin is cleared from the circulation. As a consequence, less iron is exported from the intestine and from iron stores in hepatocytes and macrophages (reviewed by Ganz and Nemeth) [23]. Hepcidin can be cleared via the kidney [24].

Various stimuli regulate hepatic hepcidin synthesis: (1) iron availability, (2) inflammatory stimuli, (3) erythropoietic demand, (4) hypoxia, and (5) endocrine signals. Table 1 provides an overview of soluble factors, receptors, signaling molecules, and transcription factors involved in the regulation of systemic iron homeostasis.

**Table 1:** Genes, proteins and receptors involved in the regulation of systemic iron homeostasis and their function.

Regulators of iron homeostasis	Abbreviation	Iron regulatory mechanisms
Activin-receptor like kinase 2	Alk2 = ACVRL	BMP Type I receptor, required for hepcidin induction under stimulated conditions. Activation leads to increased hepcidin levels [25,26,27].
Activin-receptor like kinase 3	Alk3 = BMPR1a	BMP Type I receptor, required for baseline hepcidin expression. Activation leads to hepcidin increase [25,26,28,29,30].
Activin A receptor, type IIA and II B	ActRII a and ActRIIb	BMP Type II receptors. Activation leads to hepcidin induction [31,32,33].
Bone morphogenic protein receptor 2	BMPRII	BMP Type II receptor. Activation leads to hepcidin induction [31,32,33].
Bone morphogenetic protein 6	BMP6	Agonist of the BMP receptor, ligand for the BMP-SMAD signaling pathway in cells and mice; levels increased by hepatic iron; induces hepcidin expression [34,35,36].
Bone morphogenetic protein receptor BMPER	BMPR BMPER	Receptor for BMP ligands. Induces SMAD phosphorylation, which activates a signaling cascade to stimulate hepcidin expression [25,26,28,29,30]. BMP endothelial cell precursor-derived regulator inhibits BMP signaling and decreases hepatic hepcidin expression [37,38].
Divalent metal transporter 1	DMT1	Iron transporter (Fe^2+^) in duodenal enterocytes and endosomes of most cell types [39].
Membrane-associated ferrireductase Cybrd1 (DcytB)	Cybrd1	Ferrireductase located at the apical membrane of enterocytes, reduces Fe^3+^ to Fe^2+^ [40].
Ferroportin	FPN	Iron export protein, internalized and degraded by hepcidin [20,21,22,41,42,43].
Growth and differentiation factor 15	GDF15	Possible erythropoietic-derived suppressor of hepcidin levels [44,45,46].
Hepcidin	HAMP1, Leap1	Iron regulatory hormone, synthesized mainly by the liver [12,23,47,48,49,50,51,52] (only some articles are cited here, please consider the citation index at the end of the manuscript).
*HFE*	HFE	Name of a gene mutated in the most frequent HH subtype. MHC class1-like protein involved in iron sensing; sensitizes cells to BMP stimuli; activator of hepcidin transcription [53,54,55,56,57,58,59].
Heme oxygenase-1	HO-1	Releases intracellular iron from heme [60].
Hemojuvelin	HJV	Mutation in HJV gene cause a juvenile hemochromatosis subtype, BMP co-receptor that sensitizes hepatocytes to low endogenous BMP levels and activator of hepcidin transcription [61].
Hephaestin		A multicopper oxidase homologous to ceruloplasmin, which oxidases Fe^2+^ to Fe^3+^ [11].
Interleukin-6	IL-6	Cytokine, induced by inflammation. Binds to the IL-6 receptor. Activates hepcidin expression via STAT-3 phosphorylation [62,63,64,65].
Iron regulatory protein 1 and 2	IRP-1 and IRP2	Cellular regulators of iron homeostasis that control expression of iron-regulated mRNA on a post-transcriptional level [2].
Neogenin		Interacts with HJV and BMPs, may regulate secretion of HJV and iron uptake [31,66,67].
Solute Carrier Family 11, member 2	SLC11A2	Gene encoding the divalent metal transporter 1 (DMT1 = NRAMP2 = DCT1). Iron absorption channel expressed at the brush border side of duodenal enterocytes [12,68,69,70].
	Smad 1/5/8	Signaling molecules phosphorylated by BMP receptors [71].
	Smad 4	Transcription factor that controls BMP-mediated signalling and activator of hepcidin expression [71].
	Smad 6/7	Inhibitory SMAD proteins that regulate BMP and/or TGFbeta signaling in a negative feedback manner [72].
	STAT3	Intracellular signaling molecule of the IL-6 pathway, its phosphorylation causes hepcidin induction [62,63,65,73].
Transferrin receptor 1	TFR1	Receptor for iron-bound transferrin, possibly involved in iron sensing by interacting with HFE [57,74,75].
Transferrin receptor 2	TFR2	Receptor for iron-bound transferring, possibly involved in iron sensing by interacting with HFE [76,77].
Transmembrane protease serine 6	TMPRSS6	Inhibits hepcidin expression by cleaving HJV, iron-deficiency sensor; phosphorylates Smad5 [78,79,80].
Twisted gastrulation homolog 1	TWSG1	Possible suppressor secreted from erythropoietic precursor cells to repress hepcidin levels [81].

### 1.1. Iron Availability

**Figure 2:**
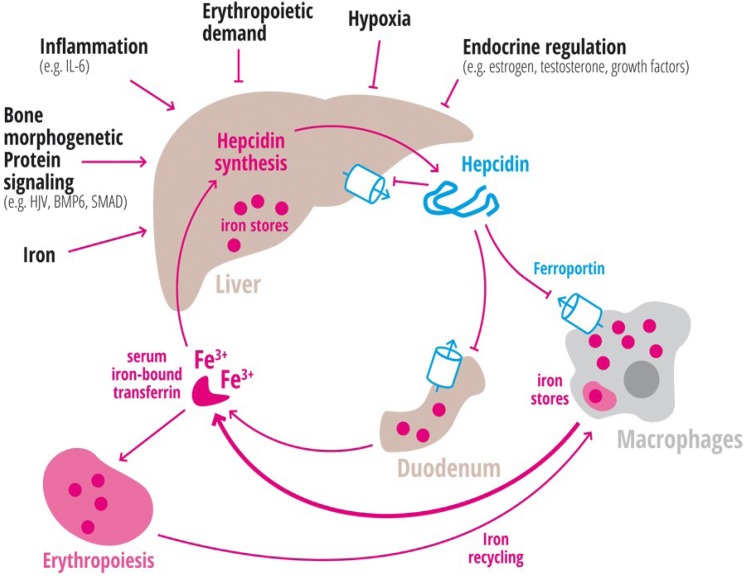
Regulation of hepatic hepcidin production. Hepatic hepcidin synthesis is regulated by iron, bone morphogenetic protein signaling, inflammation, erythropoiesis, hypoxia, or endocrine stimuli. FPN1, which is expressed predominantly in hepatocytes, macrophages and enterocytes is internalized and degraded following hepcidin binding. Iron is transported in the blood bound to transferrin. Most iron is required for erythropoiesis. Aging erythrocytes that exceed a life-span of approximately 120 days are recycled in macrophages. Transferrin-iron is a critical indicator for systemic iron homeostasis and regulator of hepcidin expression.

Tf-Fe_2_ activates hepcidin transcription in hepatocytes, which then reduces iron absorption from the diet and iron release from macrophages and hepatocytes in a negative feedback manner (Figure 2). The transcriptional response of hepcidin to iron is controlled by the bone morphogenetic protein (BMP) signaling pathway.

**Figure 3:**
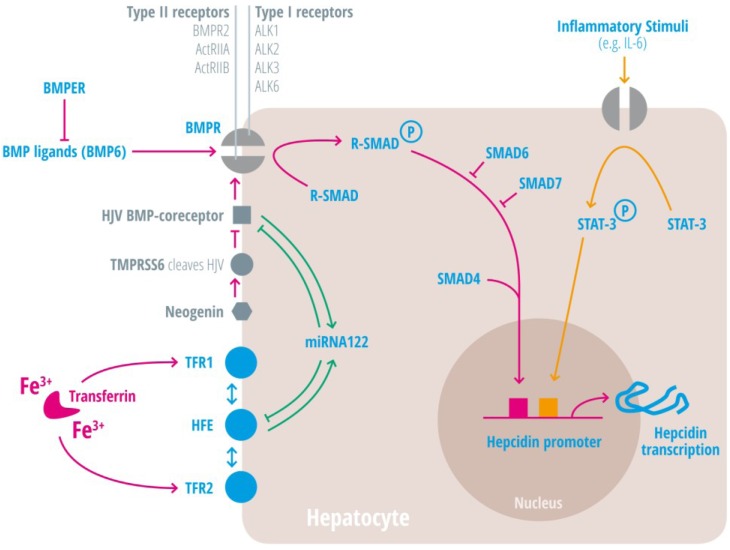
Regulation of hepatic hepcidin induction at the cellular level. Transferrin bound iron (Tf-Fe) is monitored by an “iron sensing complex”, which consists of the transferrin receptors (TfR) 1 and 2, HFE, and HJV. HJV is a glykosylphosphatidylinositol (GPI)-linked membrane associated protein that functions as a BMP coreceptor, and enhances bone morphogenetic protein (BMP) signaling. Binding of one of the more than 25 known BMP ligands (such as BMP6) to type I and II BMP receptors induces the type II receptor to phosphorylate and activate the BMP type I receptor. There are four BMP type I receptors (called ALK1, ALK2, ALK3, and ALK6), and three BMP type II receptors (BMPR2, ActRIIA, and ActRIIB). The activated BMP type I receptor leads to phosphorylation of intracellular signaling molecules called receptor associated SMAD proteins (R-SMADs). Phosphorylated R-SMADs transfer together with SMAD 4 to the hepatocyte nucleus and induce hepcidin transcription. SMAD6 and SMAD7 are inhibitory SMADs. BMPER, the BMP endothelial cell precursor-derived regulator inhibits BMP signaling and decreases hepatic hepcidin expression. MicroRNA 122 is activated by HFE or HJV and inhibits the latter in a negative feedback regulatory loop. The transmembrane serine protease (TMPRSS6) cleaves HJV and thereby decreases BMP-mediated hepcidin induction. Neogenin, a transmembrane protein known to interact with HJV, can also interact with TMPRSS6 to enable HJV cleavage in transfected cells. Soluble HJV is generated by proprotein convertase activity and has been proposed to sequester BMPs. Inflammatory stimuli such as interleukin-6 (IL-6) induce hepcidin transcription via the JAK/STAT signaling pathway. A SMAD- and a STAT-binding element have been identified in the hepcidin promoter.

Insights into iron-mediated hepcidin regulation were obtained by investigating the molecular mechanisms underlying the most frequent genetic disorder of the western world, hereditary hemochromatosis (HH). Regulated protein/protein interactions between three membrane proteins mutated in HH (the gene encoding HFE), transferrin receptor 2 (TfR2) and hemojuvelin (HJV) integrate signals elicited by the concentration of Tf-Fe_2_ and hepatocytic iron stores [60]. Because HJV is a glykosylphosphatidylinositol (GPI)-linked membrane protein that functions as a BMP co-receptor, BMP signaling was identified as the major signaling pathway that controls hepcidin regulation [61]. Bmp6, a member of the transforming growth factor-beta (TFG-β) superfamily seems to be the major ligand that activates hepcidin levels, because Bmp6 knock-out mice show severe iron overload due to a failure to activate hepcidin expression [35,82]. Binding of the ligand Bmp6 to BMP receptors type I (Alk1, Alk2, Alk3, and Alk6) and BMP receptors type II (BMPRII, ActRIIa, or ActRIIb) induces the type II receptor to phosphorylate and activate the BMP type I receptor. BMP type I receptors, that are expressed in the liver are called Alk2 and Alk3 (Figure 3) (of the other BMP type I receptors Alk1 is predominantly expressed in endothelial cells and Alk6 is not expressed in hepatocytes) [83,84]. Mice with liver-specific deficiency of Alk2 and Alk3 develop moderate to severe iron overload, respectively, due to decreased hepcidin mRNA expression [25]. Although it is known that BMP2, BMP4, and BMP6 are endogenous ligands for HJV in human hepatoma cells, and HJV uses selectively the BMP type II receptors BMPRII and ActRIIA [31], specific roles of individual BMP type II receptors in iron metabolism have yet to be investigated. The activated BMP type I receptor leads to phosphorylation of intracellular signaling molecules called receptor associated SMAD proteins (R-SMADs), which transfer together with SMAD4 to the hepatocyte nucleus to induce hepcidin transcription (Figure 3) [60]. In contrast to activation of hepcidin gene expression by Smad1/5/8 and Smad4, the inhibitory Smad6 and Smad7 decrease hepcidin in response to high iron load [72,85]. Another regulator of BMP signaling is the bone morphogenetic protein-binding endothelial cell precursor-derived regulator (Bmper), which is overexpressed in hypotransferrinemic mice (Trf(hpx/hpx)). Soluble BMPER inhibits BMP2- or BMP6-mediated hepcidin promoter activity in human HepG2 and HuH7 cells [37].

Two sequence motifs (BMP-responsive elements) are critical for hepcidin promoter activity mediated by HJV, BMP6, and SMAD4. Patients with HFE-deficiency and mice with Hfe or Tfr2-deficiency show an attenuation of BMP signaling, which suggests that these proteins control the efficiency of the BMP signaling pathway (Figure 3) [55,75]. Biochemical evidence supports these data by demonstrating that HJV, HFE, and TfR2 form a membrane associated complex in human hepatoma cells [57]. Hepcidin expression is not only controlled by signaling molecules and transcription factors but also by microRNAs (miRNA). Interestingly, the liver-expressed miRNA-122 is involved in an autoregulatory circuitry in which its high level expression depends on HFE and HJV, two target genes of miRNA-122 (Figure 3). Consistently, mice depleted of miRNA-122 show elevated mRNA levels of HFE and HJV, which cause increased hepcidin mRNA levels and plasma iron deficiency [86,87]. Additionally, miRNA-130a seems to down regulate hepcidin mRNA expression under iron deficient conditions by targeting ALK2 and SMAD5 [88].

HJV is further cleaved by the transmembrane serine protease TMPRSS6 (matriptase-2) in cells overexpressing both proteins and thereby decreases BMP-mediated hepcidin induction (Figure 3) [80]. Inactivating mutations in TMPRSS6 cause iron-refractory iron deficiency anemia (IRIDA) due to inappropriately high hepcidin levels [78,79].

TMPRSS6 expression is regulated by multiple stimuli, such as hypoxia [89,90], acute dietary iron deficiency [91], chronic high-iron diet, or by BMP6 [36], which contribute to the hepcidin response. The transmembrane protein neogenin interacts with HJV and TMPRSS6 to facilitate HJV cleavage in transfected cells (Figure 3) [66]. By contrast, mice with a homozygous mutation in neogenin are hallmarked by hepcidin deficiency and iron overload [67]. HJV is further cleaved by the proprotein convertase to generate soluble HJV (sHJV), which may sequester BMPs. sHJV was proposed to be released mainly from the skeletal muscle to modulate hepcidin expression in response to metabolic needs. However, mice with a muscle specific-deficiency of HJV could not reveal a role of sHJV in the regulation of systemic iron balance [92,93].

### 1.2. Inflammation

In the anemia of chronic disease (ACD) hepcidin expression is high and hypoferremia develops rapidly due to decreased macrophage iron release (Figure 2). If the inflammatory stimulus persists the amount of iron required for erythropoiesis is too low and anemia will develop. This is considered to represent a defense strategy against (iron-dependent) invading pathogens. Hepcidin transcription is activated by inflammatory cytokines, especially interleukin-6 (IL-6), but also others (e.g., IL-1, IL-22) (Figure 2), as well as the innate immune response to extracellular pathogens such as candida albicans or influenza A (as reviewed for example in [23,64,94,95]). IL-6 activates hepcidin by binding to the gp130 protein receptor complex, which triggers JAK1/2 (intracellular tyrosine kinase) mediated STAT3 phosphorylation. Phosphorylated STAT3 translocates to the nucleus, and activates the STAT3-binding motif of the hepcidin promoter (Figure 3) [62,63,65,94].

Several animal models have been established to partially mimic ACD: (1) IL-6 injections induce hepcidin and trigger hypoferremia in wild-type mice, but not in liver-specific SMAD4^−/−^ mice, suggesting that the BMP signaling pathway is additionally required for hepcidin activation [96]. (2) Turpentine injections into mice [26,97] and rats [29] cause sterile abscesses and induce IL-6 and hepcidin gene expression [26,73,98]. The BMP small molecule inhibitor LDN-193189, which inhibits the activity of the BMP type I receptors Alk2, Alk3, and Alk6, was able to revert hepcidin activation and ACD in turpentine treated rodents—further suggesting a cross-talk between the BMP- and the IL-6-signaling pathways [26,29]. (3) Lipopolysaccharide (LPS), a membrane constituent of gram negative bacteria is frequently injected into mice to induce hepcidin expression and hypoferremia in an IL-6-dependent manner. Blunted hepcidin responses in LPS-injected *Hfe* and *Tfr2* knock-out mice point to a functional interaction with BMP signaling [99,100]. Furthermore, injections of LPS into wild-type mice revealed a role of activin B, a member of the TGF-β superfamily, in the LPS-mediated hepcidin response [101]. (4) ACD is further mimicked by heat killed brucella abortus injections in mice, a model utilized to test anti-hepcidin antibodies for the reversion of ACD [102]. All these studies point towards a critical role of both the inflammatory JAK/STAT and the iron-related BMP signaling pathways to control the hepcidin response to inflammation. These data are supported by the analysis of the hepcidin promoter that demonstrates the necessity for both a STAT-binding site and a BMP-response element for hepcidin stimulation by IL-6 [58,63].

### 1.3. Erythropoiesis

Twenty milligrams of iron are required for erythropoiesis every day. If the iron requirements for the synthesis of new red blood cells increase (e.g., as a consequence of phlebotomy or hemolysis) hepcidin expression is inhibited to satisfy the elevated iron demand (reviewed in [23,95]). Increased erythropoiesis either as a result of phlebotomy or erythropoietin injection suppresses hepcidin [103]. Two soluble factors and members of the TGFbeta superfamily, growth and differentiation factor 15 (GDF15), and twisted gastrulation homolog 1 (TWSG1) that are released from erythroid precursor cells suppress hepcidin transcription in cellular assays and correlate with inappropriately low hepcidin levels in patients with ineffective erythropoiesis (e.g., β-thalassemia) [46,81]. However, GDF15^−/−^ mice were recently shown to respond with a decrease in hepcidin levels in response to phlebotomy similar to wild-type mice [44], suggesting that at least in mice GDF-15 does not control the hepcidin response to blood loss.

### 1.4. Hypoxia

Hypoxia stimulates erythropoietin production and erythropoiesis. As a consequence iron requirements rise and hepcidin levels decrease to allow for elevated iron absorption and release from stores (Figure 1 and Figure 2). Likewise, hypoxia caused by high altitude in humans results in diminished hepcidin levels [104,105]. HIF-1 and HIF-2 are transcription factors that are stabilized under hypoxic conditions and that regulate transcription of a large number of iron related genes (e.g., *TfR1*, *Tf*, *ceruloplasmin*, *DMT-1*, *FPN1*) (Figure 1). Experiments in mice that either lack HIF-2α or express constitutively stabilized HIF-2α (*Vhlh*/Hif-1α) suggest that HIF-2α contributes to the repression of hepcidin through erythropoietin-mediated increased erythropoiesis- but not through direct repression of hepcidin transcription [106]. By contrast, in cultured hepatocytic cells chemical HIF stabilizers suppress hepcidin mRNA directly [107]. In addition, hypoxic conditions increase transcription of TMPRSS6 [89] mediated by a hypoxic responsive element in the TMPRSS6 promoter [90]. However, the loss of *Hfe* or *Tfr2* and *Tmprss6* in genetically modified mice does not affect the hypoxic response of hepcidin [108].

### 1.5. Endocrine Regulation

More recently, growth hormones were shown to control serum iron levels and hepcidin gene expression. Hepcidin levels are increased by an extended period of fasting for three days, possibly owing to a suppression of erythropoiesis that may occur during the fasting state to preserve tissue iron concentrations [109]. In addition, administration of growth hormone decreased hepcidin levels in healthy volunteers, presumably by stimulating erythropoiesis [109]. Hepatocyte growth factor (HGF) and epidermal growth factor (EGF) suppress hepatic hepcidin synthesis, in part mediated by the PI3-kinase-MEK/ERK-kinase pathways, which modulate the nuclear localization of BMP pathway transcriptional regulators including activated Smads1/5/8 and the co-repressor transforming growth factor β-induced factor 1 (TGIF-1) [45].

Furthermore, sex hormones control hepcidin levels which may in part explain sex-specific differences in iron levels. Estradiol treatment of primary hepatocytes reduced hepcidin expression mediated by G-protein coupled receptor 30-BMP6-dependent signaling [34]. An estrogen response element (ERE) was identified in the hepcidin promoter that explains estrogen-mediated hepcidin regulation [34,110]. Likewise testosterone, which is applied to increase hemoglobin levels and to treat ACD in humans downregulates hepatic hepcidin mRNA expression by interfering with the BMP/Smad signaling pathway. Specifically, testosterone promotes the association of androgen receptor with Smad1 and Smad4 to reduce their binding to the BMP-RE in the liver in mice [111]. Furthermore, glucose increases serum hepcidin concentrations and thereby modulates serum iron [112]. Interestingly, mice heterozygous for the BMP type I receptor Alk3 (Bmpr1a) show abnormalities in glucose metabolism in an intraperitoneal glucose tolerance test [30], due to alterations of the glucose-sensing pathway and increased insulin 1 and 2 mRNA levels. Whether this affects iron metabolism has yet to be determined.

## 2. Iron Is a Critical Nutrient

Dietary iron exists in two different forms: heme and non-heme iron (Figure 1). Heme-bound iron is a constituent of hemoglobin or myoglobin and is maintained in a reduced ferrous state (Fe^2+^) for oxygen binding. High levels of heme iron are found in animal protein sources, such as red meat, fish, and poultry. Non-heme iron is maintained in an oxidized, ferric state (Fe^3+^) and is usually bound to macromolecules. The vegetarian diet mainly contains non-heme iron. Heme iron is generally absorbed better than non-heme iron, although most iron in our diet is present in the non-heme form [113,114,115]. In any case, iron absorption depends on other ingredients within food. Phytates or polyphenols, for example, inhibit iron absorption [4,116]. In a healthy individual, adequate serum iron levels can be reached no matter if iron is ingested as meat or vegetarian diet (reviewed in [117]). Iron absorption is influenced by the amount of iron present in the diet and largely depends on the composition of the ingested food, individual iron demand (e.g., for erythropoiesis) or the chemical structure of the absorbed iron. Depending on bioavailability, approximately 1 mg iron is absorbed each day. This rate can be halved if iron stores are filled, or increased to 3–5 mg/day [118]. The absorption rate can vary widely [116]; at average, men absorb around 6% and woman around 13% of their ingested iron [116].

## 3. Iron Supplementation

Most iron in the mammalian body is utilized for erythropoiesis. Thus, nutritional iron deficiency causes iron deficiency anemia, which is hallmarked by low numbers of microcytic hypoferremic erythrocytes. In addition, two markers for systemic iron availability are reduced, serum iron, and ferritin levels. As erythropoiesis has to be maintained, and iron is critical for neuronal development in the developing embryo until adolescence, iron needs to be supplemented orally or intravenously in the iron deficient state [119].

Oral iron supplementation is the preferred way of treatment, as it is easily absorbed and the formulations are cost-effective. Oral iron is applied for example as ferrous sulphate tablets (200 mg). Healthy, pregnant females additionally take 100 mg iron/day. Side effects of oral iron therapy were reported with 10% of dyspepsia, 5% constipation, and 3% diarrhea [120]. These side effects increase in severity with the amount of iron given [121]. Physicians further need to take into account interactions between the absorption of oral iron and other pharmacies such as antacids, H_2_ blockers or thyroxine. Intramuscular iron injection is no longer practiced today due to toxicity.

An alternative way to supplement iron is intravenously. Intravenous iron supplementation has a long history, and was already in practice in 1932 [122]. Bioavailability of intravenous iron is higher than of oral supplementation, and it more effectively repletes iron stores. However, as intravenous iron is stored in macrophages, enterocytes, and hepatocytes—it is critical to monitor the iron status of the patient to avoid iron toxicity.

Generally, iron administration has to be considered carefully during infections. A recent clinical study exemplified the hazardous role of iron in infection very dramatically, whereby the supplementation of iron to the diet of children in an area of high prevalence of bacterial infection and malaria (Eastern Africa) resulted in a drastic increase in the incidence and severity of bacterial meningitis and malaria along with a rise in mortality as compared to children not receiving iron [123,124]. In addition, in patients infected with human immunodeficiency virus (HIV) iron leads to higher virus replication and should not be given to these patients without critical indication [125].

## 4. Frequent Iron Related Disorders

**Figure 4:**
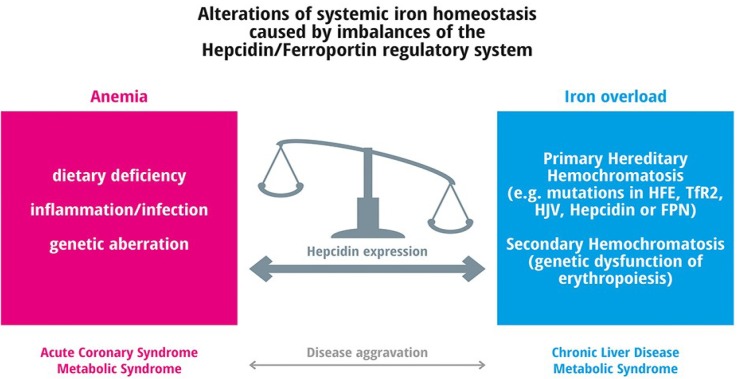
Alterations of systemic iron homeostasis caused by imbalances of the Hepcidin/Ferroportin regulatory system.

Hepcidin deregulation can be the cause of severe iron-related diseases (Figure 4). Inappropriately low hepcidin levels as observed in HH or iron-loading anemias cause iron overload, while elevated hepcidin levels, such as in anemia of chronic disease (ACD) or IRIDA, cause iron deficiency anemias [95,126]. Figure 4 demonstrates the critical role of the hepcidin/ferroportin regulatory circuitry for balancing iron homeostasis. Disturbance of systemic iron homeostasis causes two major classes of disease, anemia and hemochromatosis.

### 4.1. Anemia

According to a report published by the World Health Organization on the global burden of anemia worldwide (from 1993 to 2005), anemia is an indicator of both, poor nutrition and low health status. About 126 million people suffer from anemia worldwide [1]. Of these, only 4.9% do not have public health problems, while the majority (40.9%) is categorized with severe public health issues. Per definition, anemia occurs if the hemoglobin level drops <120 g/L for women, and <130 g/L for men, all older than 15 years of age. Generally, quality of life is impaired in anemic patients due to fatigue, dizziness, and impaired cognitive functions. The symptoms arise from iron deficiency in red blood cells which diminishes the oxygen supply for the body.

**Figure 5:**
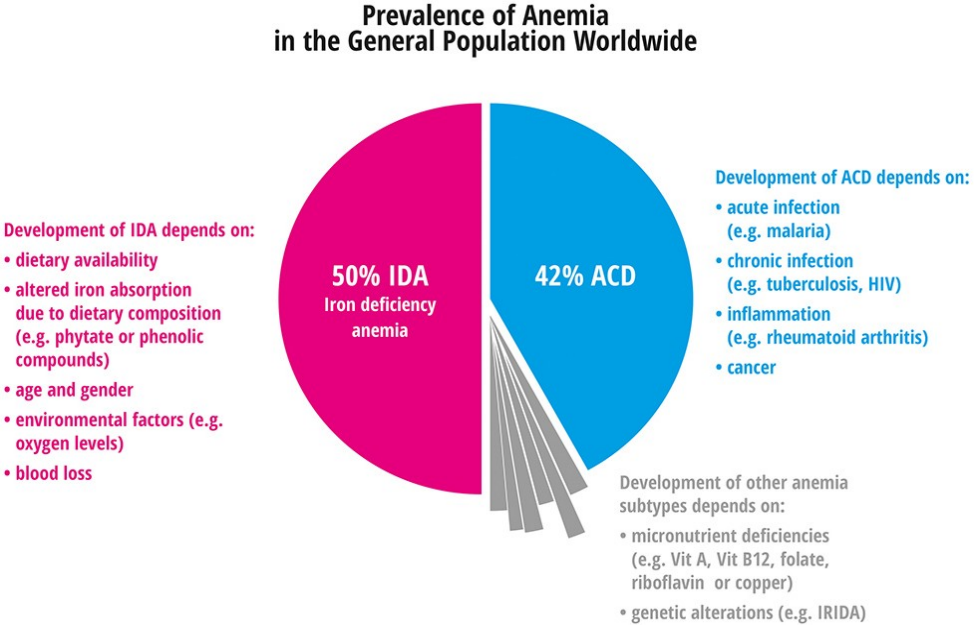
Prevalence of anemia in the general population worldwide.

Iron deficiency can occur both with or without anemia as reviewed by Denic and Agarwal [127]. It generally protects against infectious diseases such as malaria [127], but limited iron intake is associated with the aggravation of frequent diseases such as acute coronary syndrome. The prevalence of different anemia subtypes is shown in Figure 5. About 50% of anemias arise from nutritional iron deficiency, 42% are caused by inflammation and infection. The remaining 8% of anemias develop due to nutritional deficiencies (such as vitamin A, vitamin B12, folate, riboflavin, or copper), or are genetically caused. In the following paragraph details on frequent and rare anemias are listed:

#### 4.1.1. Iron Deficiency Anemia

*Iron deficiency anemia (IDA)* is the most frequent form of anemia caused by a relative or absolute deficiency of dietary iron that is insufficient to satisfy the iron demand for erythropiesis. In an early stage, iron deficiency occurs without anemia, while in later stages, anemia develops as a result of depleted iron stores. In this case, oral or intravenous iron supplementation is the treatment of choice [128,129,130]. In 2002, IDA was listed among the most important contributing factors to the global burden of disease [1].

#### 4.1.2. Anemia of Chronic Disease

*Anemia of chronic disease (ACD)* is a mild to moderate normocytic, normochromic anemia which occurs as a consequence of chronic infection, inflammation, or neoplasia. Hepcidin activation by cytokines causes low serum iron levels and transferrin saturation, but ferritin levels are high due to inflammation [64,131]. In ACD, oral iron supplementation is ineffective as elevated hepcidin levels impair intestinal iron absorption. In addition, intravenous iron only marginally improves the anemic state, as iron becomes trapped in storage sites such as macrophages and hepatocytes as a consequence of ferroportin degradation by hepcidin.

Over the past years, various therapeutic strategies have been developed, which include treatment of the underlying disease, red blood cell transfusions, erythropoietin treatment, and/or intravenous iron injections [64,132,133]. Experimentally, anti-hepcidin antibodies [102], PRS-080 [134], the “Spiegelmer” NOX-H94 [135], and inhibitors of BMP signaling, such as LDN-193189 [26,29], have been explored to decrease hepcidin levels. While LDN-193189 inhibits BMP receptors, so that hepcidin induction through the BMP receptors is blocked, anti-hepcidin antibodies and the Spiegelmers bind hepcidin, so that hepcidin cannot exert its effect on FPN. The clinical effectiveness of these compounds, especially compatibility with infections, will be focus of research within the next years.

ACD is categorized according to the underlying disease or the patient cohort presenting with ACD:

- Anemia of heart failure;
- Anemia of chronic kidney disease;
- Anemia in inflammatory rheumatic diseases;
- Anemia of the elderly.

#### 4.1.3. Myelodysplastic Syndrome

Different degrees of anemia are frequently observed in *Myelodysplastic syndrome (MDS)*, a heterogeneous group of disorders caused by genetic aberrations in genes that control hematopoiesis (reviewed recently by Garcia-Manero [136]). Clinically, MDS presents with abnormal cellular blood counts. Bone marrow biopsy and visual examination of morphological evidence of dysplasia of the bone marrow aspirate leads to diagnosis. The underlying cause of MDS is heterogeneous but mutations in the splicing factor *SF3B1* or *TET2*, a gene that functions in the control of cytosine hydroxymethylation are detected in about 50% of genetically determined MDS. Anemia hallmarks MDS as a consequence of the loss/reduction of the number of red cells and serves as a prognostic marker for disease progression. Blood transfusions are indicated to treat severe anemia. As a consequence, iron overload is also observed in MDS. It was speculated that iron overload may play a role during the transformation of MDS to acute myeloid leukaemia (AML), when ferritin levels rise above 2500 ng/mL. In this case, the use of iron chelators is recommended. A phase II study is ongoing that evaluates the role of iron chelation in MDS (NCT00940602). Curative treatment is still unavailable, so that bone marrow transplantation is the only potential cure.

#### 4.1.4. Anemias Caused by Genetic Defects

Anemias caused by genetic defects encompass a large group of rare, heterogeneous disorders. They have a prevalence of less than five cases per 10,000, and are reviewed by Gulbis *et al.* [137]. More than 80% of rare anemias follow a dominant hereditary pattern with a probability of 50% for children to inherit the disease. In 2013, 62 rare anemia subtypes were listed in the “European Network of Rare Congenital Anemias” (www.enerca.org).

Rare anemias include hemolytic anemias, such as hemoglobinopathies that are caused by inherited defects either in the structure of hemoglobin (Hb) (e.g., sickle cell disease), or in Hb synthesis (beta-thalassemia), spherocytosis that arises due to mutations in proteins responsible for the regular shape of erythrocytes or metabolic deficiencies (e.g., in glucose-6-phosphate hydrogenase) that increase oxidative stress in erythrocytes. Alterations of the shape and/or viability of erythrocytes causes hemolysis (the destruction of the red cells), which can be acute or chronic [137].

The identification of novel genes involved in iron transport and homeostasis, and availability of animal models has enabled the identification of patients with novel rare subtypes of hereditary microcytic anemias. These diseases are commonly under diagnosed and inappropriately treated due to a lack of available diagnostic strategies. These anemias arise from mutations in genes that control (1) duodenal iron absorption (e.g., DMT1 [138]; see Figure 1), (2) systemic iron homeostasis (e.g., TMPRSS6), or (3) erythroid iron absorption and utilization.

Erythroid precursor cells satisfy their high iron demand by receptor-mediated endocytosis of Tf-bound iron via TfR1. Therefore, mutations in genes involved in endosomal iron uptake or export, such as DMT-1 [139], the endosomal ferrireductase STEAP3 [140] or Sec15L1 [141,142] cause erythroblast iron deficiency and anemia. In the cytoplasm of the erythroblast iron is stored in ferritin or utilized for heme—or FeS cluster biogenesis in mitochondria. Thus, mutations in genes that interfere with mitochondrial iron transport (e.g., mitoferrin [142]) or FeS cluster biogenesis (e.g., GLRX5 [143,144], ABC7 [145,146]) can cause microcytic anemias in animal models and/or patients. For example, mutations in Alas2, the first enzyme of the heme biosynthesis pathway, cause x-linked sideroblastic anemia due to a reduction in the synthesis of PPIX (protoporphyrin IX) [147]. As a consequence, iron accumulates in erythroblasts, which triggers ROS production and cellular damage. Similarly loss of the mitochondrial glutaredoxin GLRX5 causes mitochondrial iron accumulation, oxidative stress and impaired erythrocyte function [143,144]. Thus far, a single patient with GLRX5 inactivating mutations has been described, who is hallmarked by mild microcytic anemia, iron overload, and ring sideroblasts. The Hb value dropped in the course of his life until he became transfusion dependent. Interestingly, iron chelator treatment could resolve his anemia [148].

The *iron-resistant iron deficient anemia (IRIDA)*. Genetic IRIDA is a microcytic anemia resistant to both oral and parenteral iron supplementation [149]. It is caused by mutations in the *TMPRSS6* gene encoding matriptase-2. Patients with IRIDA, and mice with impaired function of matriptase-2, show inappropriately high hepcidin levels [36,149]. TMPRSS6 is a serine protease that cleaves HJV and thereby inhibits hepcidin induction [80].

Mutations in TMPRSS6 lead to elevated hepcidin levels and ferroportin internalization and degradation. As a consequence, iron cannot be sufficiently absorbed from the intestine or released from iron stores. Therefore, iron supplementation is ineffective. Anemia is not present at birth, but develops at one month of age, and generally is more pronounced in children than in adults [149].

## 5. Iron Deficiency and Frequent Diseases

*Acute coronary syndrome (ACS)* is a leading cause of death worldwide. It occurs due to a lack of oxygen in the myocardial muscle. Anemia, either caused by nutritional iron deficiency or ACD (see Section 4.1.2.) is frequently observed in people with an increased risk to develop ACS and myocardial infarction [150]. Whether anemia causes ACS, or if anemia aggravates disease severity of ACS and myocardial infarction is poorly understood. As treatment usually has to occur quickly, iron substitution is secondary in this setting and in case of low hemoglobin levels patients receive blood transfusions.

The *metabolic syndrome* affects iron homeostasis by impairment of the hepcidin/ferroportin regulatory circuitry. In approximately one-third of patients with non-alcoholic fatty liver disease or the metabolic syndrome hyperferritinemia occurs with normal or mildly elevated transferrin saturation. Mild iron overload seems to stimulate hepcidin expression and as a consequence, FPN1 is degraded, and less iron is absorbed. Therefore, long-term obesity is frequently associated with increased hepcidin levels, iron deficiency, and anemia. Interestingly, both iron deﬁciency (particularly in severely obese patients) and iron excess (dysmetabolic iron overload syndrome), are well documented in association with obesity-related conditions and are reviewed in detail by Datz *et al.* [151].

### Iron Overload

Low hepcidin levels cause *iron overload (IO)*, also termed *hemochromatosis (HH)* in humans. HH is caused by increased iron export from enterocytes, hepatocytes and macrophages as a consequence of low hepcidin levels and ferroportin overexpression. Iron accumulates in critical organs such as the heart, liver, and pancreas where it causes oxidative stress which leads to cirrhosis, cancer, diabetes, and cardiomyopathy [152]. HH is the most frequent genetic disorder (with an allele frequency of one in eight) in people of European descent (reviewed in [152]). It develops due to mutations in genes encoding activators of hepcidin transcription (*HFE*, transferrin receptor 2 (*TFR2)*, hemojuvelin (*HFE2*, or *HJV)*, in *FPN* or in hepcidin (*HAMP)* itself [152]. The most common form of HH is caused by a mutation in the *HFE* gene.

Although the gene has already been identified in 1996, the pathophysiology has yet to be understood [56,153]. Mutations in the *HFE* gene lead to an adult onset of HH, while mutations in *TFR2*, *HJV*, and *HAMP* cause a more aggressive juvenile subtype. In HH serum iron levels, transferrin saturation and ferritin levels are increased. If HH is diagnosed early enough organ damage can be prevented by repeated phlebotomy. However, given that symptoms of HH are typically non-specific (e.g., fatigue, arthralgia, malaise, darker skin, or increase in transferrin saturation), the disease often goes undiagnosed for decades. If iron-mediated organ damage has occurred repeated phlebotomy cannot reverse the iron-induced organ damage [152].

While HH is a liver disease, secondary hemochromatosis is caused by hereditary or acquired anemia subtypes that arise from mutations in genes that lead to insufficient or malfunctioning erythrocytes (e.g., MDS, thalassemias, or sickle cell disease). In these so-called iron-loading anemias [149,154] a signal is sent from proliferating erythrocytes to the liver to decrease hepatic hepcidin levels and increase duodenal iron uptake and macrophage iron release. As the iron cannot be utilized for erythropoiesis, it accumulates in different organs causing damage. These patients frequently require blood transfusions which exacerbates the iron overload (one unit of red blood cells contains 200–250 mg iron). In this case the iron overload due to the blood transfusions is compensated for by iron chelator treatment to avoid iron toxicity [149,154,155,156].

Neonatal hemochromatosis (NH) is caused by severe fetal liver disease of the newborn with iron overload of the liver and in extrahepatic tissues. It is also called gestational alloimmune liver disease (GALD) [157]. Fetal liver controls the iron flow from the mother to the fetus to satisfy the iron demand for growth, hemoglobin synthesis and organ functions. Iron efflux from the placenta depends on FPN1. Interestingly, livers of the fetus and newborns with GALD express only low hepcidin levels compared to healthy fetuses. Thus, the liver-injury-induced failure to produce adequate amounts of hepcidin may increase the FPN1-mediated export of placental iron and explain the iron overload seen in GALD patients [157].

## 6. Iron Overload and Frequent Diseases

An increasing number of reports suggest that mild to moderate increases in tissue iron levels may have significant clinical relevance in several common, acquired disorders. These conditions include chronic liver diseases such as alcoholic (ALD) and non-alcoholic fatty liver disease (NAFLD), steatohepatitis (ASH and NASH), chronic hepatitis C infections, and end-stage liver disease [158]. In these diseases, iron deposits are detected either in hepatocytes, Kupffer/sinusoidal cells, or in both. There, proliferative and mutagenic effects of excess iron may converge to explain the pathogenic role of iron in the progression of chronic liver diseases and/or hepatocellular carcinoma development [159,160]. In addition, increased iron stores predispose to insulin resistance (while iron removal restores the response to insulin) and late diabetic complications [161,162]. Furthermore, increased body iron stores correlate with the risk for atherosclerosis and cardiovascular diseases [163]. In these disorders it was hypothesized that hepcidin promotes atherosclerosis by inducing macrophage iron accumulation and the release of cytokines. Finally, patients with Alzheimer’s disease or Parkinson’s disease experience a dramatic increase in their brain iron content which has opened the possibility that disturbances in brain iron homeostasis may contribute to the pathogenesis of these disorders [164,165,166].

## 7. Conclusions

Systemic iron homeostasis is regulated by the hepatic hormone hepcidin, which controls iron export from ferroportin expressing cell types. Dysregulation of the hepcidin/ferroportin regulatory system causes two major classes of widespread diseases: anemia in case hepcidin levels are high, or hemochromatosis in case hepcidin levels are inappropriately low. In addition, cellular iron deficiency and/or iron overload aggravates disease severity in frequent acquired diseases such as acute coronary syndrome, metabolic syndrome, or chronic liver disease.

## 8. Perspective

Systemic iron homeostasis is unbalanced in severe genetic disorders of iron metabolism and in frequent acquired diseases. The most frequent subtype of anemia is caused by nutritional iron deficiency closely followed by the anemia of chronic disease (ACD). In ACD, hepcidin levels are high due to an excess of inflammatory cytokines, which prohibits oral iron substitution. Our current understanding of regulatory mechanisms involved in systemic iron homeostasis fueled the development of novel pharmacological agents that block hepcidin induction, or hepcidin itself. Some of these are currently under evaluation in phase III studies in humans. In the future even more detailed insights into mechanisms and pathways regulating iron homeostasis will be gained by identifying novel genes mutated in genetically inherited anemias or iron overload diseases and by understanding molecular mechanisms of iron misregulation in acquired (metabolic) diseases and its pathological consequence. We expect that this will uncover clinically useful information that may lead to the development of novel therapeutic approaches or the identification of diagnostically or prognostically useful markers that can be applied to monitor disease progression.

## References

[1] McLean E., Cogswell M., Egli I., Wojdyla D., de Benoist B. (2009). Worldwide prevalence of anaemia, who vitamin and mineral nutrition information system, 1993–2005. Public Health Nutr..

[2] Muckenthaler M.U., Galy B., Hentze M.W. (2008). Systemic iron homeostasis and the iron-responsive element/iron-regulatory protein (IRE/IRP) regulatory network. Annu. Rev. Nutr..

[3] Haase V.H. (2013). Regulation of erythropoiesis by hypoxia-inducible factors. Blood Rev..

[4] Domke A., Großklaus R., Niemann B., Przyrembel H., Richter K., Schmidt E., Weißenborn A., Wörner B., Ziegenhagen R., Wissenschaft B. (2004). Utilisation of Minerals in Nutrients—Toxicologic and Nutrition-Physiologic Aspects.

[5] Gunshin H., Starr C.N., Direnzo C., Fleming M.D., Jin J., Greer E.L., Sellers V.M., Galica S.M., Andrews N.C. (2005). Cybrd1 (duodenal cytochrome b) is not necessary for dietary iron absorption in mice. Blood.

[6] Choi J., Masaratana P., Latunde-Dada G.O., Arno M., Simpson R.J., McKie A.T. (2012). Duodenal reductase activity and spleen iron stores are reduced and erythropoiesis is abnormal in Dcytb knockout mice exposed to hypoxic conditions. J. Nutr..

[7] Vanoaica L., Darshan D., Richman L., Schumann K., Kuhn L.C. (2010). Intestinal ferritin H is required for an accurate control of iron absorption. Cell Metab..

[8] Peyssonnaux C., Zinkernagel A.S., Schuepbach R.A., Rankin E., Vaulont S., Haase V.H., Nizet V., Johnson R.S. (2007). Regulation of iron homeostasis by the hypoxia-inducible transcription factors (HIFs). J. Clin. Investig..

[9] Hentze M.W. (1994). Translational control by iron-responsive elements. Adv. Exp. Med. Biol..

[10] Hentze M.W., Kuhn L.C. (1996). Molecular control of vertebrate iron metabolism: mRNA-Based regulatory circuits operated by iron, nitric oxide, and oxidative stress. Proc. Natl. Acad. Sci. USA.

[11] Vulpe C.D., Kuo Y.M., Murphy T.L., Cowley L., Askwith C., Libina N., Gitschier J., Anderson G.J. (1999). Hephaestin, a ceruloplasmin homologue implicated in intestinal iron transport, is defective in the sla mouse. Nat. Genet..

[12] Hentze M.W., Muckenthaler M.U., Galy B., Camaschella C. (2010). Two to tango: Regulation of mammalian iron metabolism. Cell.

[13] Peslova G., Petrak J., Kuzelova K., Hrdy I., Halada P., Kuchel P.W., Soe-Lin S., Ponka P., Sutak R., Becker E. (2009). Hepcidin, the hormone of iron metabolism, is bound specifically to alpha-2-macroglobulin in blood. Blood.

[14] Theurl I., Theurl M., Seifert M., Mair S., Nairz M., Rumpold H., Zoller H., Bellmann-Weiler R., Niederegger H., Talasz H. (2008). Autocrine formation of hepcidin induces iron retention in human monocytes. Blood.

[15] Nguyen N.B., Callaghan K.D., Ghio A.J., Haile D.J., Yang F. (2006). Hepcidin expression and iron transport in alveolar macrophages. Am. J. Physiol..

[16] Merle U., Fein E., Gehrke S.G., Stremmel W., Kulaksiz H. (2007). The iron regulatory peptide hepcidin is expressed in the heart and regulated by hypoxia and inflammation. Endocrinology.

[17] Kulaksiz H., Theilig F., Bachmann S., Gehrke S.G., Rost D., Janetzko A., Cetin Y., Stremmel W. (2005). The iron-regulatory peptide hormone hepcidin: Expression and cellular localization in the mammalian kidney. J. Endocrinol..

[18] Wang Q., Du F., Qian Z.M., Ge X.H., Zhu L., Yung W.H., Yang L., Ke Y. (2008). Lipopolysaccharide induces a significant increase in expression of iron regulatory hormone hepcidin in the cortex and substantia nigra in rat brain. Endocrinology.

[19] Bekri S., Gual P., Anty R., Luciani N., Dahman M., Ramesh B., Iannelli A., Staccini-Myx A., Casanova D., Ben Amor I. (2006). Increased adipose tissue expression of hepcidin in severe obesity is independent from diabetes and nash. Gastroenterology.

[20] Nemeth E., Tuttle M.S., Powelson J., Vaughn M.B., Donovan A., Ward D.M., Ganz T., Kaplan J. (2004). Hepcidin regulates cellular iron efflux by binding to ferroportin and inducing its internalization. Science.

[21] Qiao B., Sugianto P., Fung E., Del-Castillo-Rueda A., Moran-Jimenez M.J., Ganz T., Nemeth E. (2012). Hepcidin-induced endocytosis of ferroportin is dependent on ferroportin ubiquitination. Cell Metab..

[22] Ross S.L., Tran L., Winters A., Lee K.J., Plewa C., Foltz I., King C., Miranda L.P., Allen J., Beckman H. (2012). Molecular mechanism of hepcidin-mediated ferroportin internalization requires ferroportin lysines, not tyrosines or JAK-STAT. Cell Metab..

[23] Ganz T., Nemeth E. (2012). Hepcidin and iron homeostasis. Biochim. Biophys. Acta.

[24] Wolff F., Deleers M., Melot C., Gulbis B., Cotton F. (2013). Hepcidin-25: Measurement by LC-MS/MS in serum and urine, reference ranges and urinary fractional excretion. Clin. Chim. Acta.

[25] Steinbicker A.U., Bartnikas T.B., Lohmeyer L.K., Leyton P., Mayeur C., Kao S.M., Pappas A.E., Peterson R.T., Bloch D.B., Yu P.B. (2011). Perturbation of hepcidin expression by bmp type I receptor deletion induces iron overload in mice. Blood.

[26] Steinbicker A.U., Sachidanandan C., Vonner A.J., Yusuf R.Z., Deng D.Y., Lai C.S., Rauwerdink K.M., Winn J.C., Saez B., Cook C.M. (2011). Inhibition of bone morphogenetic protein signaling attenuates anemia associated with inflammation. Blood.

[27] Theurl I., Schroll A., Nairz M., Seifert M., Theurl M., Sonnweber T., Kulaksiz H., Weiss G. (2011). Pathways for the regulation of hepcidin expression in anemia of chronic disease and iron deficiency anemia *in vivo*. Haematologica.

[28] Bottcher Y., Unbehauen H., Kloting N., Ruschke K., Korner A., Schleinitz D., Tonjes A., Enigk B., Wolf S., Dietrich K. (2009). Adipose tissue expression and genetic variants of the bone morphogenetic protein receptor 1A gene (BMPR1A) are associated with human obesity. Diabetes.

[29] Theurl I., Schroll A., Sonnweber T., Nairz M., Theurl M., Willenbacher W., Eller K., Wolf D., Seifert M., Sun C.C. (2011). Pharmacologic inhibition of hepcidin expression reverses anemia of chronic inflammation in rats. Blood.

[30] Scott G.J., Ray M.K., Ward T., McCann K., Peddada S., Jiang F.X., Mishina Y. (2009). Abnormal glucose metabolism in heterozygous mutant mice for a type I receptor required for BMP signaling. Genesis.

[31] Xia Y., Babitt J.L., Sidis Y., Chung R.T., Lin H.Y. (2008). Hemojuvelin regulates hepcidin expression via a selective subset of BMP ligands and receptors independently of neogenin. Blood.

[32] Yu P.B., Beppu H., Kawai N., Li E., Bloch K.D. (2005). Bone morphogenetic protein (BMP) type II receptor deletion reveals BMP ligand-specific gain of signaling in pulmonary artery smooth muscle cells. J. Biol. Chem..

[33] Yu P.B., Deng D.Y., Beppu H., Hong C.C., Lai C., Hoyng S.A., Kawai N., Bloch K.D. (2008). Bone morphogenetic protein (BMP) type II receptor is required for BMP-mediated growth arrest and differentiation in pulmonary artery smooth muscle cells. J. Biol. Chem..

[34] Ikeda Y., Tajima S., Izawa-Ishizawa Y., Kihira Y., Ishizawa K., Tomita S., Tsuchiya K., Tamaki T. (2012). Estrogen regulates hepcidin expression via GPR30-BMP6-dependent signaling in hepatocytes. PLoS One.

[35] Meynard D., Kautz L., Darnaud V., Canonne-Hergaux F., Coppin H., Roth M.P. (2009). Lack of the bone morphogenetic protein BMP6 induces massive iron overload. Nat. Genet..

[36] Meynard D., Vaja V., Sun C.C., Corradini E., Chen S., Lopez-Otin C., Grgurevic L., Hong C.C., Stirnberg M., Gutschow M. (2011). Regulation of TMPRSS6 by BMP6 and iron in human cells and mice. Blood.

[37] Patel N., Masaratana P., Diaz-Castro J., Latunde-Dada G.O., Qureshi A., Lockyer P., Jacob M., Arno M., Matak P., Mitry R.R. (2012). Bmper protein is a negative regulator of hepcidin and is up-regulated in hypotransferrinemic mice. J. Biol. Chem..

[38] Santos P.C., Krieger J.E., Pereira A.C. (2012). Molecular diagnostic and pathogenesis of hereditary hemochromatosis. Int. J. Mol. Sci..

[39] Andrews N.C. (1999). The iron transporter DMT1. Int. J. Biochem. Cell Biol..

[40] McKie A.T., Barrow D., Latunde-Dada G.O., Rolfs A., Sager G., Mudaly E., Mudaly M., Richardson C., Barlow D., Bomford A. (2001). An iron-regulated ferric reductase associated with the absorption of dietary iron. Science.

[41] Donovan A., Lima C.A., Pinkus J.L., Pinkus G.S., Zon L.I., Robine S., Andrews N.C. (2005). The iron exporter ferroportin/Slc40a1 is essential for iron homeostasis. Cell Metab..

[42] Lymboussaki A., Pignatti E., Montosi G., Garuti C., Haile D.J., Pietrangelo A. (2003). The role of the iron responsive element in the control of ferroportin1/IREG1/MTP1 gene expression. J. Hepatol..

[43] McGregor J.A., Shayeghi M., Vulpe C.D., Anderson G.J., Pietrangelo A., Simpson R.J., McKie A.T. (2005). Impaired iron transport activity of ferroportin 1 in hereditary iron overload. J. Membr. Biol..

[44] Casanovas G., Spasic M.V., Casu C., Rivella S., Strelau J., Unsicker K., Muckenthaler M.U. (2012). The murine growth differentiation factor 15 is not essential for systemic iron homeostasis in phlebotomized mice. Haematologica.

[45] Goodnough J.B., Ramos E., Nemeth E., Ganz T. (2012). Inhibition of hepcidin transcription by growth factors. Hepatology.

[46] Tanno T., Bhanu N.V., Oneal P.A., Goh S.H., Staker P., Lee Y.T., Moroney J.W., Reed C.H., Luban N.L., Wang R.H. (2007). High levels of GDF15 in thalassemia suppress expression of the iron regulatory protein hepcidin. Nat. Med..

[47] Nicolas G., Bennoun M., Devaux I., Beaumont C., Grandchamp B., Kahn A., Vaulont S. (2001). Lack of hepcidin gene expression and severe tissue iron overload in upstream stimulatory factor 2 (USF2) knockout mice. Proc. Natl. Acad. Sci. USA.

[48] Viatte L., Lesbordes-Brion J.C., Lou D.Q., Bennoun M., Nicolas G., Kahn A., Canonne-Hergaux F., Vaulont S. (2005). Deregulation of proteins involved in iron metabolism in hepcidin-deficient mice. Blood.

[49] Pigeon C., Ilyin G., Courselaud B., Leroyer P., Turlin B., Brissot P., Loreal O. (2001). A new mouse liver-specific gene, encoding a protein homologous to human antimicrobial peptide hepcidin, is overexpressed during iron overload. J. Biol. Chem..

[50] Nicolas G., Viatte L., Bennoun M., Beaumont C., Kahn A., Vaulont S. (2002). Hepcidin, a new iron regulatory peptide. Blood Cells Mol. Dis..

[51] Nemeth E., Valore E.V., Territo M., Schiller G., Lichtenstein A., Ganz T. (2003). Hepcidin, a putative mediator of anemia of inflammation, is a type II acute-phase protein. Blood.

[52] Lee P., Peng H., Gelbart T., Beutler E. (2004). The IL-6- and lipopolysaccharide-induced transcription of hepcidin in HFE-, transferrin receptor 2-, and beta 2-microglobulin-deficient hepatocytes. Proc. Natl. Acad. Sci. USA.

[53] Corradini E., Garuti C., Montosi G., Ventura P., Andriopoulos B., Lin H.Y., Pietrangelo A., Babitt J.L. (2009). Bone morphogenetic protein signaling is impaired in an hfe knockout mouse model of hemochromatosis. Gastroenterology.

[54] Ryan J.D., Ryan E., Fabre A., Lawless M.W., Crowe J. (2010). Defective bone morphogenic protein signaling underlies hepcidin deficiency in HFE hereditary hemochromatosis. Hepatology.

[55] Bolondi G., Garuti C., Corradini E., Zoller H., Vogel W., Finkenstedt A., Babitt J.L., Lin H.Y., Pietrangelo A. (2010). Altered hepatic BMP signaling pathway in human hfe hemochromatosis. Blood Cells Mol. Dis..

[56] Feder J.N., Gnirke A., Thomas W., Tsuchihashi Z., Ruddy D.A., Basava A., Dormishian F., Domingo R., Ellis M.C., Fullan A. (1996). A novel MHC class I-like gene is mutated in patients with hereditary haemochromatosis. Nat. Genet..

[57] D’Alessio F., Hentze M.W., Muckenthaler M.U. (2012). The hemochromatosis proteins HFE, TFR2, and HJV form a membrane-associated protein complex for hepcidin regulation. J. Hepatol..

[58] Verga Falzacappa M.V., Casanovas G., Hentze M.W., Muckenthaler M.U. (2008). A bone morphogenetic protein (BMP)-responsive element in the hepcidin promoter controls HFE2-mediated hepatic hepcidin expression and its response to IL-6 in cultured cells. J. Mol. Med. (Berl.).

[59] Coppin H., Darnaud V., Kautz L., Meynard D., Aubry M., Mosser J., Martinez M., Roth M.P. (2007). Gene expression profiling of *Hfe*^−/−^ liver and duodenum in mouse strains with differing susceptibilities to iron loading: Identification of transcriptional regulatory targets of hfe and potential hemochromatosis modifiers. Genome Biol..

[60] Hentze M.W., Muckenthaler M.U., Andrews N.C. (2004). Balancing acts: Molecular control of mammalian iron metabolism. Cell.

[61] Babitt J.L., Huang F.W., Wrighting D.M., Xia Y., Sidis Y., Samad T.A., Campagna J.A., Chung R.T., Schneyer A.L., Woolf C.J. (2006). Bone morphogenetic protein signaling by hemojuvelin regulates hepcidin expression. Nat. Genet..

[62] Pietrangelo A., Dierssen U., Valli L., Garuti C., Rump A., Corradini E., Ernst M., Klein C., Trautwein C. (2007). Stat3 is required for IL-6-GP130-dependent activation of hepcidin *in vivo*. Gastroenterology.

[63] Verga Falzacappa M.V., Vujic Spasic M., Kessler R., Stolte J., Hentze M.W., Muckenthaler M.U. (2007). STAT3 mediates hepatic hepcidin expression and its inflammatory stimulation. Blood.

[64] Weiss G., Goodnough L.T. (2005). Anemia of chronic disease. N. Engl. J. Med..

[65] Wrighting D.M., Andrews N.C. (2006). Interleukin-6 induces hepcidin expression through STAT3. Blood.

[66] Enns C.A., Ahmed R., Zhang A.S. (2012). Neogenin interacts with matriptase-2 to facilitate hemojuvelin cleavage. J. Biol. Chem..

[67] Lee D.H., Zhou L.J., Zhou Z., Xie J.X., Jung J.U., Liu Y., Xi C.X., Mei L., Xiong W.C. (2010). Neogenin inhibits HJV secretion and regulates BMP-induced hepcidin expression and iron homeostasis. Blood.

[68] Muckenthaler M., Roy C.N., Custodio A.O., Minana B., deGraaf J., Montross L.K., Andrews N.C., Hentze M.W. (2003). Regulatory defects in liver and intestine implicate abnormal hepcidin and Cybrd1 expression in mouse hemochromatosis. Nat. Genet..

[69] Knutson M., Wessling-Resnick M. (2003). Iron metabolism in the reticuloendothelial system. Crit. Rev. Biochem. Mol. Biol..

[70] Montalbetti N., Simonin A., Kovacs G., Hediger M.A. (2013). Mammalian iron transporters: Families SLC11 and SLC40. Mol. Aspects Med..

[71] Wharton K.A., Serpe M. (2013). Fine-tuned shuttles for bone morphogenetic proteins. Curr. Opin. Genet. Dev..

[72] Vujic Spasic M., Sparla R., Mleczko-Sanecka K., Migas M.C., Breitkopf-Heinlein K., Dooley S., Vaulont S., Fleming R.E., Muckenthaler M.U. (2012). SMAD6 and SMAD7 are co-regulated with hepcidin in mouse models of iron overload. Biochim. Biophys. Acta.

[73] Sakamori R., Takehara T., Tatsumi T., Shigekawa M., Hikita H., Hiramatsu N., Kanto T., Hayashi N. (2010). STAT3 signaling within hepatocytes is required for anemia of inflammation *in vivo*. J. Gastroenterol..

[74] Chen J., Enns C.A. (2012). Hereditary hemochromatosis and transferrin receptor 2. Biochim. Biophys. Acta.

[75] Corradini E., Rozier M., Meynard D., Odhiambo A., Lin H.Y., Feng Q., Migas M.C., Britton R.S., Babitt J.L., Fleming R.E. (2011). Iron regulation of hepcidin despite attenuated smad1,5,8 signaling in mice without transferrin receptor 2 or HFE. Gastroenterology.

[76] Kawabata H., Yang R., Hirama T., Vuong P.T., Kawano S., Gombart A.F., Koeffler H.P. (1999). Molecular cloning of transferrin receptor 2. A new member of the transferrin receptor-like family. J. Biol. Chem..

[77] Goswami T., Andrews N.C. (2006). Hereditary hemochromatosis protein, HFE, interaction with transferrin receptor 2 suggests a molecular mechanism for mammalian iron sensing. J. Biol. Chem..

[78] Finberg K.E., Heeney M.M., Campagna D.R., Aydinok Y., Pearson H.A., Hartman K.R., Mayo M.M., Samuel S.M., Strouse J.J., Markianos K. (2008). Mutations in TMPRSS6 cause iron-refractory iron deficiency anemia (IRIDA). Nat. Genet..

[79] Beutler E., van Geet C., te Loo D.M., Gelbart T., Crain K., Truksa J., Lee P.L. (2010). Polymorphisms and mutations of human TMPRSS6 in iron deficiency anemia. Blood Cells Mol. Dis..

[80] Silvestri L., Pagani A., Nai A., de Domenico I., Kaplan J., Camaschella C. (2008). The serine protease matriptase-2 (TMPRSS6) inhibits hepcidin activation by cleaving membrane hemojuvelin. Cell Metab..

[81] Tanno T., Porayette P., Sripichai O., Noh S.J., Byrnes C., Bhupatiraju A., Lee Y.T., Goodnough J.B., Harandi O., Ganz T. (2009). Identification of TWSG1 as a second novel erythroid regulator of hepcidin expression in murine and human cells. Blood.

[82] Andriopoulos B., Corradini E., Xia Y., Faasse S.A., Chen S., Grgurevic L., Knutson M.D., Pietrangelo A., Vukicevic S., Lin H.Y. (2009). BMP6 is a key endogenous regulator of hepcidin expression and iron metabolism. Nat. Genet..

[83] Otu H.H., Naxerova K., Ho K., Can H., Nesbitt N., Libermann T.A., Karp S.J. (2007). Restoration of liver mass after injury requires proliferative and not embryonic transcriptional patterns. J. Biol. Chem..

[84] Yu P.B., Hong C.C., Sachidanandan C., Babitt J.L., Deng D.Y., Hoyng S.A., Lin H.Y., Bloch K.D., Peterson R.T. (2008). Dorsomorphin inhibits bmp signals required for embryogenesis and iron metabolism. Nat. Chem. Biol..

[85] Mleczko-Sanecka K., Casanovas G., Ragab A., Breitkopf K., Muller A., Boutros M., Dooley S., Hentze M.W., Muckenthaler M.U. (2010). SMAD7 controls iron metabolism as a potent inhibitor of hepcidin expression. Blood.

[86] Castoldi M., Muckenthaler M.U. (2012). Regulation of iron homeostasis by microRNAs. Cell. Mol. Life Sci..

[87] Castoldi M., Vujic Spasic M., Altamura S., Elmen J., Lindow M., Kiss J., Stolte J., Sparla R., D’Alessandro L.A., Klingmuller U. (2011). The liver-specific microRNA miR-122 controls systemic iron homeostasis in mice. J. Clin. Investig..

[88] Zumbrennen-Bullough K., Wu Q., Chen W., Babitt J. MicroRNA-130a Downregulates Hepcidin Expression during Iron Deficiency by Targeting ALK2. Proceedings of Fifth Congress of the International BioIron Society (IBIS), Biennial World Meeting (BioIron 2013).

[89] Lakhal S., Schodel J., Townsend A.R., Pugh C.W., Ratcliffe P.J., Mole D.R. (2011). Regulation of type II transmembrane serine proteinase TMPRSS6 by hypoxia-inducible factors: New link between hypoxia signaling and iron homeostasis. J. Biol. Chem..

[90] Maurer E., Gutschow M., Stirnberg M. (2012). Matriptase-2 (TMPRSS6) is directly up-regulated by hypoxia inducible factor-1: Identification of a hypoxia-responsive element in the TMPRSS6 promoter region. Biol. Chem..

[91] Zhang A.S., Anderson S.A., Wang J., Yang F., DeMaster K., Ahmed R., Nizzi C.P., Eisenstein R.S., Tsukamoto H., Enns C.A. (2011). Suppression of hepatic hepcidin expression in response to acute iron deprivation is associated with an increase of matriptase-2 protein. Blood.

[92] Chen W., Huang F.W., de Renshaw T.B., Andrews N.C. (2011). Skeletal muscle hemojuvelin is dispensable for systemic iron homeostasis. Blood.

[93] Gkouvatsos K., Wagner J., Papanikolaou G., Sebastiani G., Pantopoulos K. (2011). Conditional disruption of mouse HFE2 gene: Maintenance of systemic iron homeostasis requires hepatic but not skeletal muscle hemojuvelin. Hepatology.

[94] Armitage A.E., Eddowes L.A., Gileadi U., Cole S., Spottiswoode N., Selvakumar T.A., Ho L.P., Townsend A.R., Drakesmith H. (2011). Hepcidin regulation by innate immune and infectious stimuli. Blood.

[95] Finberg K.E. (2013). Regulation of systemic iron homeostasis. Curr. Opin. Hematol..

[96] Wang R.H., Li C., Xu X., Zheng Y., Xiao C., Zerfas P., Cooperman S., Eckhaus M., Rouault T., Mishra L. (2005). A role of SMAD4 in iron metabolism through the positive regulation of hepcidin expression. Cell Metab..

[97] Prince O.D., Langdon J.M., Layman A.J., Prince I.C., Sabogal M., Mak H.H., Berger A.E., Cheadle C., Chrest F.J., Yu Q. (2012). Late stage erythroid precursor production is impaired in mice with chronic inflammation. Haematologica.

[98] Fattori E., Cappelletti M., Costa P., Sellitto C., Cantoni L., Carelli M., Faggioni R., Fantuzzi G., Ghezzi P., Poli V. (1994). Defective inflammatory response in interleukin 6-deficient mice. J. Exp. Med..

[99] Roy C.N., Custodio A.O., de Graaf J., Schneider S., Akpan I., Montross L.K., Sanchez M., Gaudino A., Hentze M.W., Andrews N.C. (2004). An HFE-dependent pathway mediates hyposideremia in response to lipopolysaccharide-induced inflammation in mice. Nat. Genet..

[100] Wallace D.F., McDonald C.J., Ostini L., Subramaniam V.N. (2011). Blunted hepcidin response to inflammation in the absence of HFE and transferrin receptor 2. Blood.

[101] Besson-Fournier C., Latour C., Kautz L., Bertrand J., Ganz T., Roth M.P., Coppin H. (2012). Induction of activin B by inflammatory stimuli up-regulates expression of the iron-regulatory peptide hepcidin through Smad1/5/8 signaling. Blood.

[102] Sasu B.J., Cooke K.S., Arvedson T.L., Plewa C., Ellison A.R., Sheng J., Winters A., Juan T., Li H., Begley C.G. (2010). Antihepcidin antibody treatment modulates iron metabolism and is effective in a mouse model of inflammation-induced anemia. Blood.

[103] Pak M., Lopez M.A., Gabayan V., Ganz T., Rivera S. (2006). Suppression of hepcidin during anemia requires erythropoietic activity. Blood.

[104] Piperno A., Galimberti S., Mariani R., Pelucchi S., Ravasi G., Lombardi C., Bilo G., Revera M., Giuliano A., Faini A. (2011). Modulation of hepcidin production during hypoxia-induced erythropoiesis in humans *in vivo*: Data from the HIGHCARE project. Blood.

[105] Talbot N.P., Lakhal S., Smith T.G., Privat C., Nickol A.H., Rivera-Ch M., Leon-Velarde F., Dorrington K.L., Mole D.R., Robbins P.A. (2012). Regulation of hepcidin expression at high altitude. Blood.

[106] Liu Q., Davidoff O., Niss K., Haase V.H. (2012). Hypoxia-inducible factor regulates hepcidin via erythropoietin-induced erythropoiesis. J. Clin. Investig..

[107] Braliou G.G., Verga Falzacappa M.V., Chachami G., Casanovas G., Muckenthaler M.U., Simos G. (2008). 2-Oxoglutarate-dependent oxygenases control hepcidin gene expression. J. Hepatol..

[108] Lee P., Hsu M.H., Welser-Alves J., Peng H. (2012). Severe microcytic anemia but increased erythropoiesis in mice lacking hfe or TFR2 and TMPRSS6. Blood Cells Mol. Dis..

[109] Troutt J.S., Rudling M., Persson L., Stahle L., Angelin B., Butterfield A.M., Schade A.E., Cao G., Konrad R.J. (2012). Circulating human hepcidin-25 concentrations display a diurnal rhythm, increase with prolonged fasting, and are reduced by growth hormone administration. Clin. Chem..

[110] Hou Y., Zhang S., Wang L., Li J., Qu G., He J., Rong H., Ji H., Liu S. (2012). Estrogen regulates iron homeostasis through governing hepatic hepcidin expression via an estrogen response element. Gene.

[111] Guo W., Bachman E., Li M., Roy C.N., Blusztajn J., Wong S., Chan S.Y., Serra C., Jasuja R., Travison T.G. (2013). Testosterone administration inhibits hepcidin transcription and is associated with increased iron incorporation into red blood cells. Aging Cell.

[112] Aigner E., Felder T.K., Oberkofler H., Hahne P., Auer S., Soyal S., Stadlmayr A., Schwenoha K., Pirich C., Hengster P. (2013). Glucose acts as a regulator of serum iron by increasing serum hepcidin concentrations. J. Nutr. Biochem..

[113] Hallberg L. (1983). Iron requirements and bioavailability of dietary iron. Experientia.

[114] Hallberg L., Hulthen L., Garby L. (1998). Iron stores in man in relation to diet and iron requirements. Eur. J. Clin. Nutr..

[115] Hunt J.R. (2003). Bioavailability of iron, zinc, and other trace minerals from vegetarian diets. Am. J. Clin. Nutr..

[116] Yip R. (2001). Chapter 30. Iron. Present Knowledge in Nutrition.

[117] Lonnerdal B. (2009). Soybean ferritin: Implications for iron status of vegetarians. Am. J. Clin. Nutr..

[118] Cook J.D. (1990). Adaptation in iron metabolism. Am. J. Clin. Nutr..

[119] Polin V., Coriat R., Perkins G., Dhooge M., Abitbol V., Leblanc S., Prat F., Chaussade S. (2013). Iron deficiency: From diagnosis to treatment. Dig. Liver Dis..

[120] Sharma J.B., Jain S., Mallika V., Singh T., Kumar A., Arora R., Murthy N.S. (2004). A prospective, partially randomized study of pregnancy outcomes and hematologic responses to oral and intramuscular iron treatment in moderately anemic pregnant women. Am. J. Clin. Nutr..

[121] Pena-Rosas J.P., De-Regil L.M., Dowswell T., Viteri F.E. (2012). Daily oral iron supplementation during pregnancy. Cochrane Database Syst. Rev..

[122] Heath C., Strauss M., Castle W. (1932). Quantitative aspects of iron deficiency in hypochromic anemia. (The parenteral administration of iron). J. Clin. Investig..

[123] Righetti A.A., Adiossan L.G., Ouattara M., Glinz D., Hurrell R.F., N’Goran E.K., Wegmuller R., Utzinger J. (2013). Dynamics of anemia in relation to parasitic infections, micronutrient status, and increasing age in South-Central Côte d’ivoire. J. Infect. Dis..

[124] Haji K.A., Khatib B.O., Smith S., Ali A.S., Devine G.J., Coetzee M., Majambere S. (2013). Challenges for malaria elimination in Zanzibar: Pyrethroid resistance in malaria vectors and poor performance of long-lasting insecticide nets. Parasit. Vectors.

[125] Drakesmith H., Prentice A. (2008). Viral infection and iron metabolism. Nat. Rev..

[126] Zhang X., Rovin B.H. (2013). Beyond anemia: Hepcidin, monocytes and inflammation. Biol. Chem..

[127] Denic S., Agarwal M.M. (2007). Nutritional iron deficiency: An evolutionary perspective. Nutrition.

[128] Brugnara C. (2003). Iron deficiency and erythropoiesis: New diagnostic approaches. Clin. Chem..

[129] Clark S.F. (2008). Iron deficiency anemia. Nutr. Clin. Pract..

[130] Swanson C.A. (2003). Iron intake and regulation: Implications for iron deficiency and iron overload. Alcohol.

[131] Weinstein D.A., Roy C.N., Fleming M.D., Loda M.F., Wolfsdorf J.I., Andrews N.C. (2002). Inappropriate expression of hepcidin is associated with iron refractory anemia: Implications for the anemia of chronic disease. Blood.

[132] Roy C.N. (2010). Anemia of inflammation. Hematology Am. Soc. Hematol. Educ. Program.

[133] Weiss G., Gordeuk V.R. (2005). Benefits and risks of iron therapy for chronic anaemias. Eur. J. Clin. Investig..

[134] Hohlbaum A., Gille H., Christian J., Allerdsodrfer A., Jaworski J., Burrows J., Rattenstetter B., Kolodziejczyk M., Olwill S., Audoly L. Iron Mobilization and Pharmacodynamic Marker Measurements in Non-Human Primates Following Administration of PRS-080, a Novel and Highly Specific Anti-Hepcidin Therapeutic. Proceedings of Fifth Congress of the International BioIron Society (IBIS), Biennial World Meeting (BioIron 2013).

[135] Schwoebel F., van Eijk L.T., Zboralski D., Sell S., Buchner K., Maasch C., Purschke W.G., Humphrey M., Zollner S., Eulberg D. (2013). The effects of the anti-hepcidin spiegelmer NOX-H94 on inflammation-induced anemia in cynomolgus monkeys. Blood.

[136] Garcia-Manero G. (2012). Myelodysplastic syndromes: 2012 Update on diagnosis, risk-stratification, and management. Am. J. Hematol..

[137] Gulbis B., Eleftheriou A., Angastiniotis M., Ball S., Surralles J., Castella M., Heimpel H., Hill A., Corrons J.L. (2010). Epidemiology of rare anaemias in europe. Adv. Exp. Med. Biol..

[138] Iolascon A., de Falco L. (2009). Mutations in the gene encoding DMT1: Clinical presentation and treatment. Semin. Hematol..

[139] Bardou-Jacquet E., Island M.L., Jouanolle A.M., Detivaud L., Fatih N., Ropert M., Brissot E., Mosser A., Maisonneuve H., Brissot P. (2011). A novel N491s mutation in the human SLC11A2 gene impairs protein trafficking and in association with the G212V mutation leads to microcytic anemia and liver iron overload. Blood Cells Mol. Dis..

[140] Sendamarai A.K., Ohgami R.S., Fleming M.D., Lawrence C.M. (2008). Structure of the membrane proximal oxidoreductase domain of human Steap3, the dominant ferrireductase of the erythroid transferrin cycle. Proc. Natl. Acad. Sci. USA.

[141] Zhang A.S., Sheftel A.D., Ponka P. (2006). The anemia of “Haemoglobin-deficit” (hbd/hbd) mice is caused by a defect in transferrin cycling. Exp. Hematol..

[142] Troadec M.B., Warner D., Wallace J., Thomas K., Spangrude G.J., Phillips J., Khalimonchuk O., Paw B.H., Ward D.M., Kaplan J. (2011). Targeted deletion of the mouse mitoferrin1 gene: From anemia to protoporphyria. Blood.

[143] Ye H., Jeong S.Y., Ghosh M.C., Kovtunovych G., Silvestri L., Ortillo D., Uchida N., Tisdale J., Camaschella C., Rouault T.A. (2010). Glutaredoxin 5 deficiency causes sideroblastic anemia by specifically impairing heme biosynthesis and depleting cytosolic iron in human erythroblasts. J. Clin. Investig..

[144] Bergmann A.K., Campagna D.R., McLoughlin E.M., Agarwal S., Fleming M.D., Bottomley S.S., Neufeld E.J. (2010). Systematic molecular genetic analysis of congenital sideroblastic anemia: Evidence for genetic heterogeneity and identification of novel mutations. Pediatr. Blood Cancer.

[145] Allikmets R., Raskind W.H., Hutchinson A., Schueck N.D., Dean M., Koeller D.M. (1999). Mutation of a putative mitochondrial iron transporter gene (ABC7) in X-linked sideroblastic anemia and ataxia (XLSA/A). Hum. Mol. Genet..

[146] Savary S., Allikmets R., Denizot F., Luciani M.F., Mattei M.G., Dean M., Chimini G. (1997). Isolation and chromosomal mapping of a novel ATP-binding cassette transporter conserved in mouse and human. Genomics.

[147] Balwani M., Desnick R.J. (2012). The porphyrias: Advances in diagnosis and treatment. Blood.

[148] Camaschella C., Campanella A., de Falco L., Boschetto L., Merlini R., Silvestri L., Levi S., Iolascon A. (2007). The human counterpart of zebrafish shiraz shows sideroblastic-like microcytic anemia and iron overload. Blood.

[149] Camaschella C., Poggiali E. (2011). Inherited disorders of iron metabolism. Curr. Opin. Pediatr..

[150] Lawler P.R., Filion K.B., Dourian T., Atallah R., Garfinkle M., Eisenberg M.J. (2013). Anemia and mortality in acute coronary syndromes: A systematic review and meta-analysis. Am. Heart J..

[151] Datz C., Felder T.K., Niederseer D., Aigner E. (2013). Iron homeostasis in the metabolic syndrome. Eur. J. Clin. Investig..

[152] Pietrangelo A. (2011). Hereditary hemochromatosis: Pathogenesis, diagnosis, and treatment. Gastroenterology.

[153] Babitt J.L., Lin H.Y. (2011). The molecular pathogenesis of hereditary hemochromatosis. Semin. Liver Dis..

[154] Musallam K.M., Cappellini M.D., Wood J.C., Taher A.T. (2012). Iron overload in non-transfusion-dependent thalassemia: A clinical perspective. Blood Rev..

[155] Adams R.L., Bird R.J. (2013). Safety and efficacy of deferasirox in the management of transfusion-dependent patients with myelodysplastic syndrome and aplastic anaemia: A perspective review. Ther. Adv. Hematol..

[156] Thuret I. (2013). Post-transfusional iron overload in the haemoglobinopathies. C. B. Biol..

[157] Whitington P.F. (2012). Gestational alloimmune liver disease and neonatal hemochromatosis. Semin. Liver Dis..

[158] Pietrangelo A. (2009). Iron in NASH, chronic liver diseases and HCC: How much iron is too much?. J. Hepatol..

[159] Nelson J.E., Wilson L., Brunt E.M., Yeh M.M., Kleiner D.E., Unalp-Arida A., Kowdley K.V. (2011). Relationship between the pattern of hepatic iron deposition and histological severity in nonalcoholic fatty liver disease. Hepatology.

[160] Lambrecht R.W., Sterling R.K., Naishadham D., Stoddard A.M., Rogers T., Morishima C., Morgan T.R., Bonkovsky H.L. (2011). Iron levels in hepatocytes and portal tract cells predict progression and outcome of patients with advanced chronic hepatitis C. Gastroenterology.

[161] Ruivard M., Laine F., Ganz T., Olbina G., Westerman M., Nemeth E., Rambeau M., Mazur A., Gerbaud L., Tournilhac V. (2009). Iron absorption in dysmetabolic iron overload syndrome is decreased and correlates with increased plasma hepcidin. J. Hepatol..

[162] Martinelli N., Traglia M., Campostrini N., Biino G., Corbella M., Sala C., Busti F., Masciullo C., Manna D., Previtali S. (2012). Increased serum hepcidin levels in subjects with the metabolic syndrome: A population study. PLoS One.

[163] Valenti L., Dongiovanni P., Motta B.M., Swinkels D.W., Bonara P., Rametta R., Burdick L., Frugoni C., Fracanzani A.L., Fargion S. (2011). Serum hepcidin and macrophage iron correlate with MCP-1 release and vascular damage in patients with metabolic syndrome alterations. Arterioscler. Thromb. Vasc. Biol..

[164] Altamura S., Muckenthaler M.U. (2009). Iron toxicity in diseases of aging: Alzheimer’s disease, Parkinson’s disease and atherosclerosis. J. Alzheimers Dis..

[165] Liu B., Moloney A., Meehan S., Morris K., Thomas S.E., Serpell L.C., Hider R., Marciniak S.J., Lomas D.A., Crowther D.C. (2011). Iron promotes the toxicity of amyloid beta peptide by impeding its ordered aggregation. J. Biol. Chem..

[166] Lee H.P., Zhu X., Liu G., Chen S.G., Perry G., Smith M.A., Lee H.G. (2010). Divalent metal transporter, iron, and Parkinson’s disease: A pathological relationship. Cell Res..

